# Unblocking Blockbusters: Using Boolean Text-Mining to Optimise Clinical Trial Design and Timeline for Novel Anticancer Drugs

**DOI:** 10.4137/cin.s2666

**Published:** 2009-08-17

**Authors:** Richard J. Epstein

**Affiliations:** Laboratory of Computational Oncology, Department of Medicine, The University of Hong Kong, Hong Kong

**Keywords:** bibliometrics, medical informatics, drug development, clinical trials, oncology

## Abstract

Two problems now threaten the future of anticancer drug development: (i) the information explosion has made research into new target-specific drugs more duplication-prone, and hence less cost-efficient; and (ii) high-throughput genomic technologies have failed to deliver the anticipated early windfall of novel first-in-class drugs. Here it is argued that the resulting crisis of blockbuster drug development may be remedied in part by innovative exploitation of informatic power. Using scenarios relating to oncology, it is shown that rapid data-mining of the scientific literature can refine therapeutic hypotheses and thus reduce empirical reliance on preclinical model development and early-phase clinical trials. Moreover, as personalised medicine evolves, this approach may inform biomarker-guided phase III trial strategies for noncytotoxic (antimetastatic) drugs that prolong patient survival without necessarily inducing tumor shrinkage. Though not replacing conventional gold standards, these findings suggest that this computational research approach could reduce costly ‘blue skies’ R&D investment and time to market for new biological drugs, thereby helping to reverse unsustainable drug price inflation.

## Introduction

The development of massive online text archives and biomedical data warehouses over the last decade has created new opportunities to mine useful information from fuzzy datasets comprising both statistical and semantic information.^1^–^3^ Association rules have been developed to extend fuzzy set theory to text-mining; at the simplest level, identification of a given set (e.g. both A and B not C) at higher-than-expected frequency provides support for the probability of a causal link between A and B in the absence of C, albeit with varying confidence levels quantifiable as membership functions or degrees of truth. The non-digital algorithms so derived may generate value from masses of noisy data, in effect representing Bayesian interpretations of semantic information.^4^ Examples include tracking of influenza epidemics from search engine patterns,^5^ the epidemiologic surveillance of AIDS,^6^ monitoring of the blogosphere,^7^ academic publication analysis,^8^ and assessment of scientific merit based on impact factors.^9^

The causality of such associations gleaned from text-mining of the oncology literature is readily illustrated *a posteriori*. Based on existing knowledge we predict, for example, that the association of the string {papillomavirus} with {cervical cancer} will be greater than with {nasopharyngeal cancer}, whereas the reverse will be true for {Epstein-Barr virus}. Similarly, we may infer that lung cancer is more lethal than breast cancer, given that the texts {lung cancer} and {mortality} associate 50% more often than {breast cancer} and {mortality}, whereas the association between these diseases and {incidence} is similar (χ^2^ = 1059.99, df = 1, *p* < 0.01; Supplement S1). The “efficient citation hypothesis” so implied can be used to test research strategies that are becoming impractically slow and/or expensive to validate using historical approaches.

New anticancer drug development, by virtue of its target-specific nature, should readily lend itself to such semi-digital analysis.^10^ Returns on investment in applied cancer research have been declining in recent years due to a decline of blockbuster drug frequency.^11^ This has led in turn to progressive escalation of the cost for bringing a therapeutic to market, which now approaches US$1bn per FDA-licensed product;^12^ such expense is in turn handed on to the health-care consumer.^13^ Part of this cost relates to the inefficiency of the modern clinical trial system, for which problem many investigators are seeking fresh approaches such as multi-arm designs,^14^ personalized medicine or pharmacodiagnostics.^15^ A related problem specific to the cancer field is that selection of drugs for costly phase III trials remains based exclusively on demonstration of drug ‘activity’ quantified as tumor shrinkage, or response.^16^ This endpoint, although convincingly associated with short-term therapeutic benefit, may lack sensitivity for detecting the metastasis-inhibiting activity of non-tumorilytic drugs—which could well correlate strongly with survival benefit, and be better predicted by biomarker expression.^17^ Hence, the present study seeks to test how low-cost text-mining may improve applied cancer research feasibility while reducing investment risk.

## Methods

Searches were undertaken using the latest text-based search-and-retrieval version of PubMed—a service of the National Library of Medicine developed by the National Center for Biotechnology Information used to integrate the major databases (including PubMed Central, Journals, Books, OMIM). The Entrez cross database search page was used to access the Entrez Global Query database search engine. A search across the Entrez database was performed by entering one or more search term(s) or phrase(s) to execute the search.

Using this approach, the PubMed database was serially interrogated using the terms representing both the primary set of interest (P) and the secondary sets of interest (S1, S2, etc.), resulting in identification of the common set of interest (C1, C2, etc.). Arithmetical correction was made for different frequencies of S1, S2, etc., permitting calculation of the expected value of C for a given S intersection given knowledge of previous values of C and assuming the null hypothesis. The ratio of C2 to C1 was then considered the multiplier (M2) by which the null hypothesis for S2 (compared to S1) was tested. Non-parametric statistical analysis was performed by chi-square calculation.

## Results

**Figure 1:**
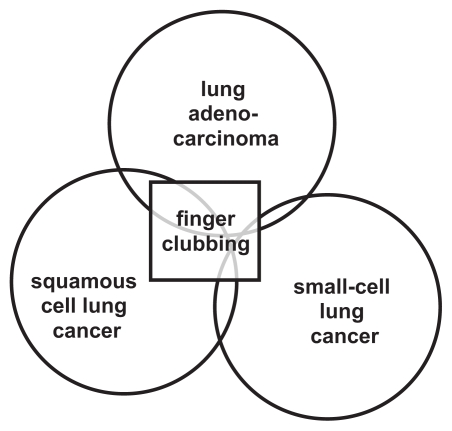
Schematic illustration of a text-mined association. The extent to which a phenotype such as finger clubbing is associated with a specific lung cancer histology may be estimated by association (see Supplement S2), as visualized here.

An initial example of how disease biology or phenotype can be predicted by text-mining is presented in Figure 1 and Supplement S2, which illustrate that the clinical complication of finger clubbing is more often associated with lung tumors of either squamous cell carcinoma or adenocarcinoma histology than with small-cell tumors (χ^2^ = 37.96, df = 2, *p* < 0.01). Similarly, it is possible to show that {brain metastasis} text in breast cancer is 80-fold more strongly associated with tumor HER2 expression than with ER expression (χ^2^ = 73.461, df = 1, *p* < 0.01; Supplement S3A). By the same token, {peritoneal carcinomatosis} is more often associated with primary invasive lobular cancers than with invasive ductal cancers (χ^2^ = 18.75, df = 1, *p* < 0.01), as is the molecule {E-cadherin} (χ^2^ = 92.98, df = 1, *p* < 0.01; Supplement S3B), expression of which is known to be selectively lost in the former tumor type. In contrast, there is no significant difference between the frequency of association of {E-cadherin} and {peritoneal metastases} (χ^2^ = 0.42, df = 1, *p* = 0.5169; Supplement S3B), consistent with the possibility that these molecular and clinical terms are causal co-variables. Such text associations can thus generate or support hypotheses concerning biomarkers in a way that may be relevant to designing therapeutic strategies in clinical research.

**Figure 2:**
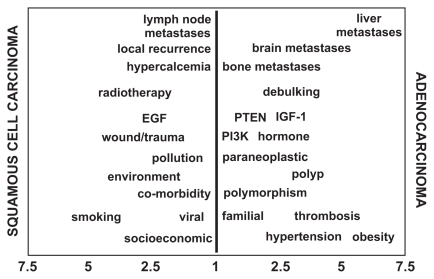
Phenotypic distinction of ‘adenocarcinoma’ vs. ‘squamous carcinoma’ using text-mining; for each phenotype shown, the PubMed database was exclusively interrogated (e.g. adenocarcinoma NOT squamous cell carcinoma, and vice versa). The figures on the horizontal axis represent the multiplier (M) by which each phenotype is preferentially associated with the histology labelled on the adjacent vertical axis.

Molecular biological issues may also be addressed by text-mining which shows, for example, that the p53 signalling (apoptotic) pathway is 70% more strongly associated with the *ARF* gene of the two-gene *CDKN2A* locus than is its *INK4A* partner (χ^2^ =63.19, df =1, *p* <0.01; Supplement S4); this suggests that these two sub-genes mediate distinct cell choices relating to cell death vs. cell-cycle delay/repair, which could prove relevant to biomarker development. Such an approach can be extended to mapping multiple phenotypes, both clinical and molecular, as depicted in Figure 2; the ‘word picture’ so created by desktop text-mining is used here to polarize the phenotypes of two otherwise generic clinical entities, adenocarcinoma and squamous cell carcinoma.

This strategy can be further applied to questions relating to clinical trial design. Imagine, for example, that there are two novel candidate anticancer therapeutics: one is an antagonist to insulin-like growth factor-1 (IGF1), and the other an inhibitor of signalling via the hepatocyte growth factor (HGF, scatter factor, c-met ligand) pathway. A simple text-mining search shows that HGF is eight-fold more strongly associated with tumor metastasis, whereas IGF1 is three times more strongly associated with drug resistance (χ^2^ = 564.15, df = 1, *p* < 0.01; Supplement S5). Such data may reasonably suggest to an oncologist that clinical trials of the HGF antagonist should test the hypothesis that it reduces metastasis in one or more tissue sites—either by reducing time to progression and/or improving overall survival, perhaps when used as a maintenance or adjuvant therapy^18^—rather than judging it solely on the basis of tumor shrinkage in progressive metastatic disease (see below). The IGF1 inhibitor, on the other hand, may be best used concurrently with chemotherapy as a sensitizer in metastatic or neoadjuvant settings, in which contexts tumor shrinkage may be a sensitive measure of its value.

Table 1 provides another illustration of hypothesis generation relevant to clinical trials using similar secondary data sets. In this case, a company has drugs of two classes, either tyrosine kinase inhibitors (TKIs) or metalloprotease inhibitors (MPIs). The question is again raised: is there any difference in the respective likelihoods that one drug class will be more active in causing tumor responses (i.e. antagonizing replication) while the other may be less active in its antimitogenic action but more active in suppressing metastasis? As shown, the text-mined association of MPIs with antimetastatic (vs. antiproliferative) key words is 6.5-fold that of TKIs, whereas the reverse trend is true for antiproliferative terms (χ^2^ = 2099.6, df = 1, *p* < 0.01); the multiplicative difference of the two trends is about 15-fold (as is true for the strings {kinase} and {protease} alone, χ^2^ = 4889.64, df = 1, *p* < 0.01; Supplement S6). Hence, one could reasonably hypothesise that clinical trials evaluating the anticancer efficacy of protease inhibitors may (i) best evaluate parameters relevant to metastasis prevention (e.g. time to progression) rather than response, and (ii) use maintenance/adjuvant rather than induction schedules in their clinical trial designs.

This approach could also be applied to prediction of targeted drug efficacy for different disease subtypes prior to the advent of a validated biomarker. Consider the scenario in which a company has a promising mTOR pathway inhibitor and is wanting to prioritise its phase 2–3 clinical trial strategy. Since the Akt proto-oncogene is an important node in the mTOR signalling network, we may begin by characterising the text-mined associations of different tumor subtypes with Akt; this analysis shows that the strongest association is with prostate cancer (Table 2). In contrast, when assessing the associations of the mTOR pathway inhibitor temsirolimus, the strongest association is with its licensed indication in renal cell carcinoma. Although not surprising, this difference raises the commercially relevant hypothesis that a ‘second use’ for mTOR inhibitors could well be confirmed for not limited to, prostate cancer.

**Figure 3:**
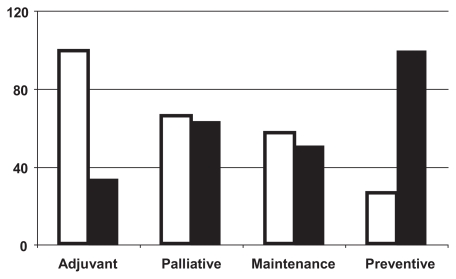
Text-mined comparison of survival vs. cost data for different cancer treatment modes, suggesting that adjuvant therapies are most strongly linked to survival benefit (open columns) but least to cost-effectiveness (solid columns), whereas preventive treatments have the opposite associations. Palliative and maintenance treatments appear intermediate for both secondary endpoints.

The associations of different treatment approaches with text-mined costs and benefits may also be compared using this seamless approach. Figure 3 shows the comparative quantitative associations of the secondary variable {overall survival benefit) with the primary variables of either {adjuvant cancer therapy} in column 1, {palliative cancer therapy} in column 2, {maintenance cancer therapy} in column 3, or {preventive cancer therapy} in column 4, and reveals a steady decline in that order. In contrast, this trend is reversed when {cost-effectiveness} is substituted as the secondary variable. This striking difference suggests that third-party payers could scrutinize the value of adjuvant treatment claims more intensively, while subsidizing preventive interventions for healthy individuals with greater enthusiasm. For companies under pressure to prove survival benefits for product licensing, on the other hand, these data suggest that drugs used in palliative or maintenance settings may deliver more easily defended ratios of QALY to unit cost than adjuvant studies (as well as being cheaper to conduct in the first instance). The possibility is also raised that biologically-targeted preventive agents could become the backbone of the blockbuster drug market.

## Discussion

The central finding of this study is an exciting one for modern researchers: namely, that the sudden availability of huge online text-based databases permits novel, cheap and potentially important research to be conducted by computer-based researchers, thereby expanding the scope and accessibility of research methodology. Moreover, by extending this approach, innovative informatics strategies may be developed and automated over time to derive value from otherwise wasted informational resources. This is true not only for curiosity-driven academic research but, as shown here, also for commercially-driven applied science.

It must be emphasized, however, that numerous pitfalls await the unwary who seek to ascribe causality to text associations without rigorous exclusion of confounders (to appreciate this, one has only to consider the famous ‘beer-and-diapers’ market research anecdote).^19^ The challenges include unambiguous identification of genes or other molecules, leading to false-positive data inclusions, and failure of current text-mining systems to achieve 100% recall, leading to false-negative loss of sensitivty. Hence, unless carefully excluded, synonyms and name variations may cause significant the accuracy of text-based data mining.

An additional problem relates to system ‘noise’ which, in any text-mined database, may be expected to increase as the age of the data archive increases. To address the confounding effect of ageing data will involve inevitable compromises between database size and data novelty; on the other hand, more recent data may be subject to dispute or lack of replication, so may be less reliable than more durable (and hence, text-repetitive) data. A further limitation is that text terms may change over time, with molecules or drugs being renamed, or having different names in different languages or countries. Such limitations require prior knowledge on the part of the investigator(s) to enable corrective action in anticipation.

How can a purely text-mining approach distinguish positive and negative associations? A given article examining two variables may not report a simple positive association, but rather the lack of an association or even an inverse or negative association; text-mining will not distinguish these contingencies, however. In general, however, it is reasonable to assume that positive associations will vastly outnumber negative associations when measured by text-mining, if only because negative associations tend to be less interesting to readers (and hence to journals and publishers) who may in general be keener to understand causality than exceptions or confounders.

Notwithstanding these important caveats, it is becoming clear that improved knowledge management is essential to regaining momentum in pharmaceutical R&D,^20^ and this unmet need requires fresh approaches. The advantages of the text-mining approach described here are that it is simple, cheap, problems fast, objective, and powerful. Its disadvantages are at least as plentiful, but perhaps the main ones are (i) that it is easily confounded by overlooking nonrandom associations which may not be obvious to all investigators, and (ii) that careful thought and broad background knowledge are needed to frame a series of questions that can provide a useful association. These limitations are sufficiently serious to restrict the use of such approaches for now to hypothesis generation, but it seems reasonable to expect that methodologic refinement and practical experience will lead to far more sophisticated approaches that will eventually be incorporated into routine research practice. Indeed, it may even become possible to devise programs that scan scientific literature and experimental data in the background, generating associations and hypotheses at different thresholds of certainty and improbability (creativity). In some ways this would be an expected outcome of the information explosion which now produces more data than any individual can hope to absorb and evaluate.^21^

The examples given here relate to the field of oncology, but could just as readily be applied to other therapeutic areas such as cardiovascular or neurological disease. In the same way that credit card companies and search engines now scan electronic data for commercially useful patterns, so can scientists now begin looking beyond their own laboratory benches in an effort to exploit the informatic power of modern databases. Although the challenges of text-data specificity we may discover that the fruits of the genomic revolution will be greater than any of us can yet conceive.

## Supplementary Data

Table S1 Data upon which the conclusions in paragraph 2 of the Introduction are based.

**Table:** 

		AND incidence	AND mortality
Breast cancer	(197552)	39175	19733
Lung cancer	(166982)	33320 (39318)	24871 (29348)
M		1.18 (NS)	1.49

Text associations suggesting that lung cancer is a significantly more lethal disease than is breast cancer (χ^2^ = 1059.99, df = 1, *p* < 0.01).

Table S2 Text data upon which figure 1 is based.

**Table:** 

	AND		AND		M
Lung cancer	Squamous cell carcinoma	16493	Finger clubbing	54	3.27
	Small-cell carcinoma	34255		42	1.23
	Adenocarcinoma	28885		101	3.50

Text-mining data showing that {small-cell lung cancer} is significantly less often associated with {finger clubbing) than are the other two histologic labels (χ^2^ = 37.96, df = 2, *p* < 0.01).

Table S3A Text-mined data alluded to in the text of para 1, Results. Text association of HER2 expression in breast cancer with brain metastasis.

**Table:** 

	AND *HER2-positive*		AND *ER-positive*
*Breast cancer*	473		2037
AND *Brain metastases*	19		1
M1	4.02		0.05
M2		80.4	

Data set showing a significantly stronger association of {brain metastases} in {breast cancer} associated with the term {HER2-positive} than with {ER-positive) (χ^2^ = 73.461, df **=** 1, p < 0.01).

Table S3B Association of lobular breast cancer histology and {E-cadherin} text string with peritoneal metastasis.

**Table:** 

	AND *invasive ductal*	AND *invasive lobular*
*Breast cancer*	13310	4649
AND *Peritoneal metastases*	28	30
*Breast cancer*	13310	4649
AND *E-cadherin*	243	209
*E-cadherin*	243	209
AND *Peritoneal metastases*	28	30

χ^2^ = 18.75, df = 1, p < 0.01.

χ^2^ = 92.98, df = 1, p < 0.01.

χ^2^ = 0.42, df = 1, p = 0.5169.

Data showing stronger association of {invasive lobular cancer} with {peritoneal metastases} than with {invasive ductal cancer}, while implicating {E-cadherin} as a molecular co-variable of this clinical relationship (see text).

Table S4 Text-mined data showing the association between *ARF* and p53, compared to that between *INK4A* and p53.

**Table:** 

*CDKN2A*	AND *INK4A*	AND *ARF*
No p53	4794	772
+p53	1568	429

Text-mined data showing a significantly stronger association of {p53} with {*ARF*} than with {*INK4A*}. (χ^2^ = 63.19, df = 1, *p* < 0.01).

Table S5 Data suggesting a stronger association between HGF and metastasis, and between IGF-1 and drug resistance.

**Table:** 

	Insulin-like growth factor-1	Hepatocyte growth factor
Drug resistance	319	100
Metastasis	100	815

Data showing significantly stronger association of {metastasis} with {HGF} than with {IGF1}, but stronger association of {drug resistance} with IGF1 than with HGF (χ^2^ = 564.15, df = 1, *p* < 0.01).

Table S6 Text comparison of ‘kinase’ and ‘protease’ strings as an association of pro-replicative vs. pro-metastatic terminology (see also Table 1).

**Table:** 

	AND (growth OR replication OR mitosis)	AND (migration OR motility OR metastasis)
Kinase	147941	23748
Protease	44028	15732

Quantification of text-mined molecular phenotype: there is a greater than 16-fold difference in the association of ‘kinase’ with pro-mitogenic vs. pro-metastatic key words when compared with the corresponding associations of ‘protease’ (χ^2^ = 4889.64, df = 1, *p* < 0.01).

## Figures and Tables

**Table 1:** Text-mined associative phenotyping of drug action. TKIs are more strongly associated with {growth and replication} than MPIs, whereas the reverse appears true of {invasion and metastasis} (χ^2^ = 2099.6, df = 1, *p* < 0.01).

	AND (growth OR replication)	AND (invasion OR metastasis)
Tyrosine kinase inhibitor (*n* = 20118)	10741	1010
Metalloprotease inhibitor (*n* = 12790)	3063	1877

**Table 2:** Association of tumor subtypes with a putative surrogate biomarker for the mTOR pathway, Akt, showing that the strongest association is with prostate cancer. As expected, the much stronger correlation of the mTOR inhibitor temsirolimus, which is already licensed for use in renal cell cancer, is with the latter malignancy. P, primary set size; C, intersecting set size.

	Breast cancer	Renal cancer	Colorectal cancer	Prostate cancer	Lung cancer	Endometrial cancer
P	196883	75450	117408	78484	166465	20194
AND *Akt*	1208	152	349	719	638	117
(100 × C)	0.61	0.20	0.30	0.92	0.38	0.58
AND *temsirolimus*	26	129	3	8	3	3
(100 × C)	0.01	0.17	0.003	0.01	0.002	0.015
